# Seroprevalence of Anti-Rubella and Anti-Measles IgG Antibodies in Pregnant Women in Shiraz, Southern Iran: Outcomes of a Nationwide Measles-Rubella Mass Vaccination Campaign

**DOI:** 10.1371/journal.pone.0055043

**Published:** 2013-01-31

**Authors:** Behnam Honarvar, Mohsen Moghadami, Afagh Moattari, Amir Emami, Neda Odoomi, Kamran Bagheri Lankarani

**Affiliations:** 1 Health Policy Research Center, Shiraz University of Medical Sciences, Shiraz, Iran; 2 HIV/AIDS Research Center, Shiraz University of Medical Sciences, Shiraz, Iran; 3 Department of Virology, School of Medicine, Shiraz University of Medical Sciences, Shiraz, Iran; 4 Health Department, Shiraz University of Medical Sciences, Shiraz, Iran; Health Protection Agency, United Kingdom

## Abstract

**Objective:**

Nonimmune pregnant women are at risk of developing congenital rubella syndrome and measles complications. We aimed to identify pregnant women susceptible to rubella or measles in order to determine the need for immunity screening and supplemental immunization in women of childbearing age.

**Method:**

This seroprevalence survey was conducted by convenience sampling in obstetric hospitals affiliated with Shiraz University of Medical Sciences (southern Iran). Serum IgG levels were measured by ELISA.

**Result:**

Mean age of the 175 pregnant women was 27.3±5.3 (range 16 to 42) years. The geometric mean concentration of anti-rubella IgG was 14.9 IU/mL (CI 95%,14.1–15.5), and that of anti-measles IgG was 13.8 IU/mL (CI 95%, 13–14.5). One hundred sixty-eight women (96%) had a protective serologic level (>11 IU/mL) of IgG against rubella, and 143 (81.7%) had a protective level against measles. Except for a significant inverse correlation that was showed by univariate analysis between anti-rubella IgG and the women’s age (P = 0.01), immunity did not correlate with demographic or obstetric characteristics or medical history. There was no significant correlation between anti-rubella and anti-measles IgG levels (P = 0.25).

**Conclusion:**

Nearly a decade after Iran’s nationwide measles-rubella vaccination campaign for the population aged 5–25 years, most pregnant women up to 34 years of age had humoral immunity against rubella. We recommend rubella immunity screening or catch-up immunization for women older than 35 years who wish to become pregnant, and measles immunity screening and appropriate vaccination for all women of childbearing age.

## Introduction

Rubella is a teratogenic virus [1] and congenital rubella syndrome (CRS) is an important cause of severe birth defects. When a woman acquires rubella virus early in pregnancy, she has a 90% chance of passing the virus on to her fetus. This can cause the death of the fetus, and may cause CRS [2]. Among vaccine-preventable diseases, measles is the leading cause of death with an estimated 450 deaths each day worldwide [3]. If measles occurs during the late stages of pregnancy, maternal and fetal morbidity will be increased. These pregnant women are exposed to a higher risk of miscarriage, severe respiratory distress, pneumonitis, hospital admission and death. Fetal death, prematurity and subacute sclerosing panencephalitis are also seen more often in infants of these women [4].

Before the introduction of rubella vaccine, the incidence of CRS varied from 0.1 to 0.2/1000 live births during endemic periods, and from 0.8 to 4/1000 live births during rubella epidemics. However, rubella vaccination during the past decade has drastically reduced or practically eliminated rubella and CRS in many developed countries and in some developing countries [2]. The precise prevalence of CRS in Iran is not known, but it was measured indirectly and estimated to be 0.2/1000 live births before rubella vaccination [5]. As rubella control progresses towards elimination, the sensitivity and specificity of surveillance systems should increase, and if resources permit, periodic seroprevalence surveys can be used to monitor the impact of immunization programs. These surveys could include women attending antenatal clinics [6]. The World Health Organization (WHO) recommends that countries undertaking measles elimination should use the opportunity to eliminate rubella as well through the use of measles-rubella (MR) or measles-mumps-rubella (MMR) vaccination in childhood immunization programs and campaigns [7]. According to the Expanded Immunization Program, in Iran all 12- and 18-month-old infants are immunized with MMR vaccine, but in response to the increased numbers of cases in older age groups during 1996–2002, a nationwide MR vaccination campaign was conducted in December 2003, and 33.5 million persons (99%) aged 5 to 25 years were vaccinated [8].

However, nearly one decade later, the lack of a routine screening system to detect women of childbearing age at risk for rubella before marriage or conception has led to a gap in our knowledge: it is unknown whether the level of immunity against rubella in women who participated in the 2003 campaign has changed significantly. Moreover, pregnant women who are now 35 years of age or older were not among the target groups in the 2003 MR mass vaccination program. Meanwhile, the mean age of marriage and first pregnancy has increased in Iran, and legal abortion for rubella infection during pregnancy faces many restrictions. The present study was therefore designed to estimate the number of pregnant women at risk of developing CRS or measles complications. Evidence from this study will help to inform future strategies for rubella and measles immunity screening or supplemental immunization activities among women of childbearing age before marriage or conception.

## Patients and Methods

This cross-sectional and questionnaire-based seroprevalance study was conducted from November 2011 to January 2012 at obstetrics and maternity hospitals affiliated with Shiraz University of Medical Sciences in Shiraz, the capital city of Fars province in southern Iran. A convenience sampling method was used. The sample size necessary for this study was 138 pregnant women, calculated with the formula z^2^Pq/d^2^ and assuming that P = 90%, confidence level = 95%, and error = 5%. The prevalence of immunity level (90%) was extracted from earlier research that determined this to be the approximate rate of immunity in pregnant women against measles and rubella [9].

There were no exclusion criteria except for refusal to participate. Two major obstetrics and maternity hospitals (Hafez and Zeinabieh Hospitals) are affiliated with Shiraz University of Medical Sciences; most pregnant women in Shiraz seek prenatal care at these centers. Initially, three trained, experienced midwives explained the aims of the study to the women and completed the informed consent forms and questionnaires. A 10-mL blood sample was taken from each participant. Each woman was interviewed face to face privately, and blood samples were obtained separately from each of them at the obstetrics clinics at each of the two participating hospitals.

The questionnaire consisted of an introductory explanation about the aims of the study and the identity of the researchers, followed by questions regarding demographic and obstetric characteristics of the participants and their medical history. The content and face validity of the questionnaire were evaluated by expert opinion, and its reliability was calculated as 0.65 according to the Kuder-Richardson formula 20 (KR_20_).

Body mass index was calculated as weight (kg) divided by height per square meter (m^2^). Pregnant women with a gestational age (documented by their last menstrual period or pelvic sonography) of up to 14 weeks, 14^+1^ to 28 weeks, and 28^+1^ weeks were categorized as being in their first, second or third trimester of pregnancy, respectively. Blood samples were transferred promptly to the virology reference laboratory of Shiraz University of Medical Sciences, with appropriate safeguards to maintain cold chain standards. Serum IgG level was measured with an enzyme-linked immunosorbent assay (ElISA) using Ig G single test-Varicell kit, for measles (G/M 1001) and for rubella (G 1026 ). There is some variation among different studies regarding the concentration of IgG antibodies that is considered to be protective against rubella or measles. Some studies consider the presence of rubella IgG antibodies ≥10 IU/mL to provide evidence of protection [2], but in our study and in accordance with the protocol used by the virology department of Shiraz University of Medical Sciences, we categorized IgG levels against rubella or measles in pregnant women into three groups: nonimmune (<9 IU/mL), equivocal (9–11 IU/mL) or immune (>11 IU/mL).

All data were entered to SPSS version 11.5 software (SPSS, Chicago, IL, USA). The accuracy of data entry was ensured by randomly selecting and checking completed questionnaires against their corresponding data in the SPSS software. Graphs were produced with Microsoft Office Excel 2007. Differences between immune and nonimmune groups in demographic and obstetric characteristics and history of medical diseases were determined with the nonparametric chi-squared or Fischer’s exact test as appropriate. Correlations of interval and nominal variables with anti-rubella and anti-measles IgG antibody levels were determined by the Pearson and Eta coefficients, respectively. Simultaneous correlation of the most important variables (P≤0.20) as independent variables and IgG level as the dependent variable was determined by forward logistic regression. P values less than 0.05 were considered significant.

### Ethics Approval

The Ethics Committee of the Health Policy Research Center affiliated with Shiraz University of Medical Sciences approved conducting this study based on the protocol described in the patients and methods. We obtained written consent of all participants in this study. The content of consent forms was approved by Ethics Committee of the Health Policy Research Center and consisted of introductory brief explanation about aims and steps of this study, including 10 mL blood withdrawal of them, and identifications of interviewers for interviewees. The signed consent forms were kept in archive of records of this survey. All potential participants who declined to participate or otherwise did not participate were eligible for treatment (if applicable) and were not disadvantaged in any other way by not participating in the study.

## Results

One hundred seventy-five pregnant women participated in this study. Their mean age was 27.3±5.3 years (median 27, range from 16 to 42). One hundred sixty women (91.4%) were 34 years old or younger. All except four pregnant women up to 34 years of age had a positive history of MR vaccination during Iran’s nationwide 2003 mass vaccination campaign, but none of the women 35 years old or older had been vaccinated. One hundred twenty-six women (72%) resided in urban areas and 49 (28%) in rural areas. One hundred sixty-five (94.2%) were unemployed. All women had a high school level of education except for 12 (6.8%) who had graduated from university and 20 (11.4%) who had only primary school education. Thirty-eight women (21.7%) had a history of abortion or stillbirth in previous pregnancies. One hundred sixty-four (93.7%) had a singleton fetus and 5 (2.8%) had multiple fetuses in the current pregnancy. In 85 (48.5%) pregnancies the fetus was male and in 41 (23.4%) the fetus was female. One pregnant woman had both a male and female fetus, and in 48 (27.4%) pregnancies the gender of the fetus was undetermined. The number of persons in the household ranged from 2 to 11 (median 3, mean 3.3±1.7) and the number of children ranged from 0 to 7 (median 0, mean 0.6±0.8). Mean body mass index before pregnancy was 24.3±4.3, and for women who were in their first trimester, mean body mass index was 28.4±4.7. Fifty-seven (32.5%) women had history of at least one medical disease such as urinary tract infection in 49 (28%), anemia in 41 (23.4%), menstrual disorders before the current pregnancy in 19 (10.8%), diabetes mellitus in 12 (6.8%), hypertension in 8 (4.5%), psychological disease in 8 (4.5%), and asthma, cardiovascular diseases or hypothyroidism in 5 women (2.8%). Another 5 (2.8%) women were on corticosteroid treatment. Four (2.2%) women were in their first trimester of pregnancy, 62 (35.4%) were in their second trimester and 105 (60%) were in their third trimester; in 4 (2.2%) women, gestational age was not determined. Mean gestational age was 29.6±6.5 weeks (median 30, range 11 to 39). Mean time since the previous pregnancy was 3.8±3.6 years (median 3, range 0 to 16).

The geometric mean concentration of anti-rubella IgG was 14.9 IU/mL (CI 95%,14.1–15.5), and this concentration for anti-measles IgG was 13.8 IU/mL (CI 95%, 13–14.5). One hundred sixty-eight (96%; 93.1% to 98.9%) of the women had a protective level (>11 IU/mL) of anti-rubella IgG compared to 5 (2.8%; 0.4% to 5.2%) who had an equivocal (9–11 IU/mL) and 2 (1.1%; 0.4% to 2.6%) who had a nonprotective (<9 IU/mL) level of immunoglobulin. One hundred forty-three (81.7%; 79.9% to 83.5%) of the women had a protective level (>11 IU/mL) of anti-measles IgG compared to 15 (8.5%; 4.4% to 12.6%) who had an equivocal (9–11 IU/mL) and 17 (9.7%; 5.4% to 14%) who had a nonprotective (<9 IU/mL) level of immunoglobulin.

**Figure 1:**
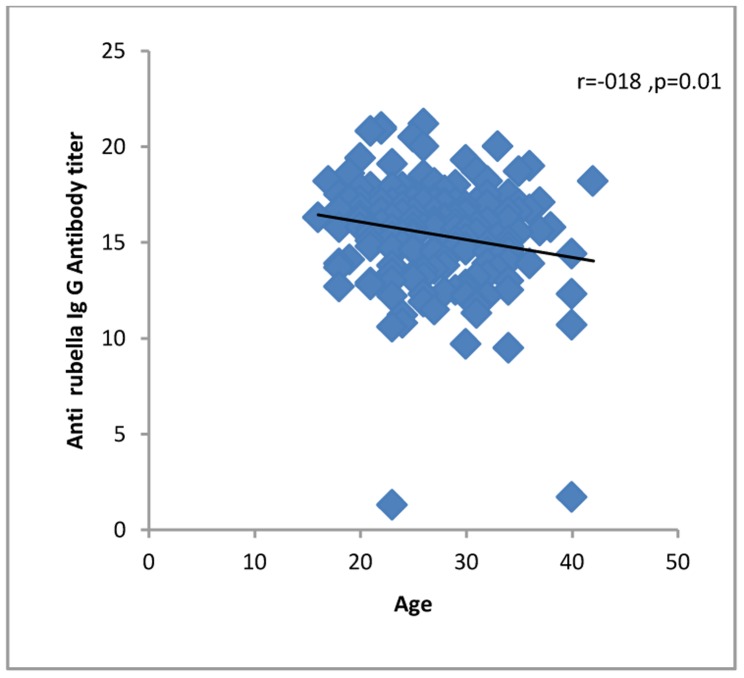
Correlation of age with anti-rubella IgG antibody level in pregnant women who sought prenatal care at obstetrics and maternity hospitals affiliated with Shiraz University of Medical Sciences in southern Iran, from November 2011 to January 2012.

**Figure 2:**
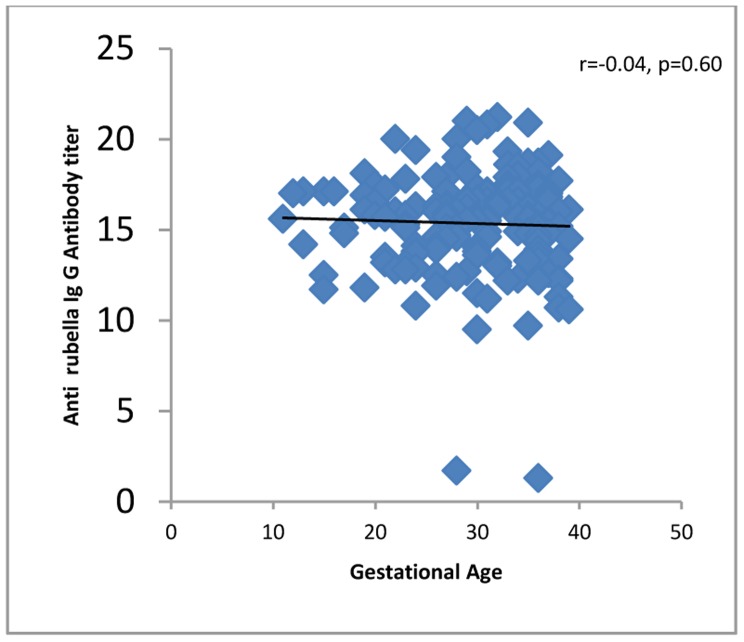
Correlation of gestational age with anti-rubella IgG antibody level in pregnant women who sought prenatal care at obstetrics and maternity hospitals affiliated with Shiraz University of Medical Sciences in southern Iran, from November 2011 to January 2012.

**Figure 3:**
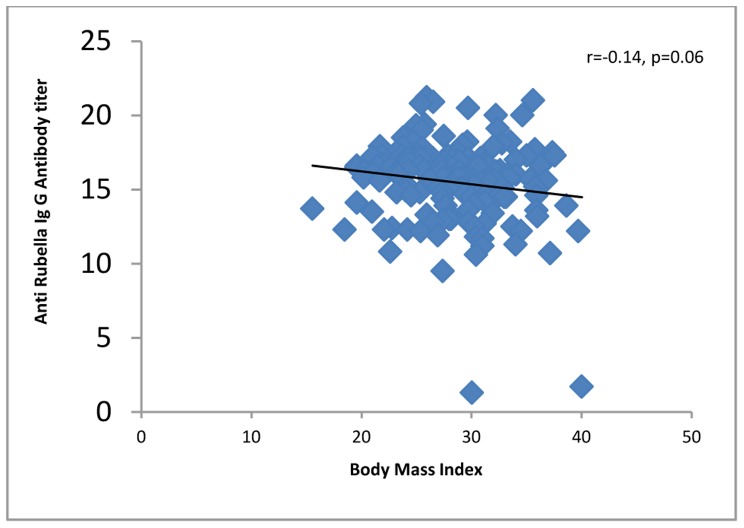
Correlation of first trimester body mass index with anti-rubella IgG antibody level in pregnant women who sought prenatal care at obstetrics and maternity hospitals affiliated with Shiraz University of Medical Sciences in southern Iran, from November 2011 to January 2012.

**Figure 4:**
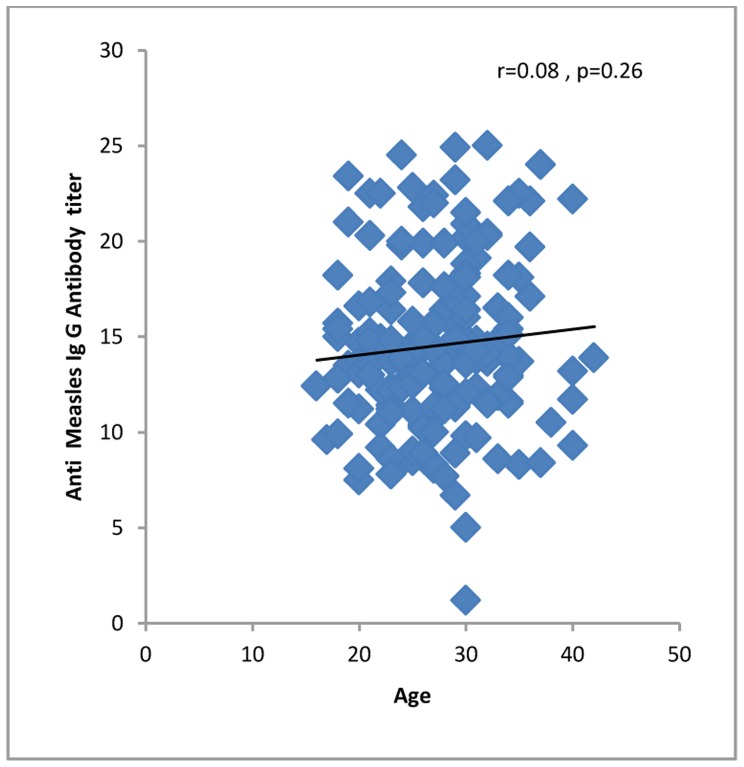
Correlation of age with anti-measles IgG antibody level in pregnant women who sought prenatal care at obstetrics and maternity hospitals affiliated with Shiraz University of Medical Sciences in southern Iran from November 2011 to January 2012.

**Figure 5:**
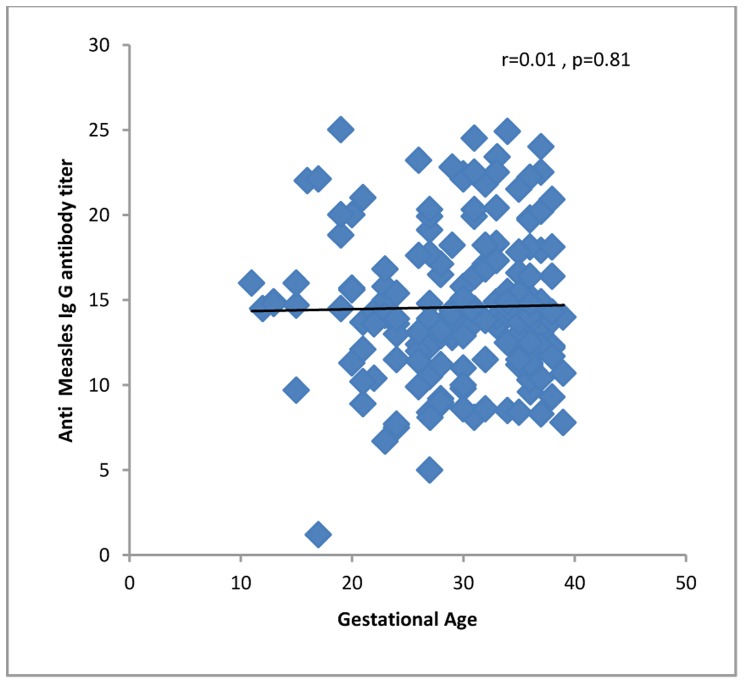
Correlation of gestational age with anti-measles IgG antibody level in pregnant women who sought prenatal care at obstetrics and maternity hospitals affiliated with Shiraz University of Medical Sciences in southern Iran from November 2011 to January 2012.

**Figure 6:**
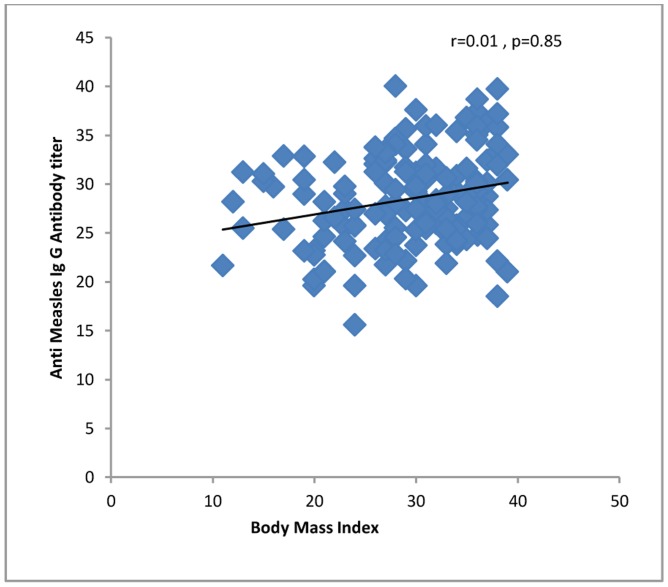
Correlation of first trimester body mass index with anti-measles IgG antibody level in pregnant women who sought prenatal care at obstetrics and maternity hospitals affiliated with Shiraz University of Medical Sciences in southern Iran from November 2011 to January 2012.

All 4 (100%) of the women in their first trimester of pregnancy were immune against rubella compared to 60 (96.7%) second trimester and 100 (95.2%) third trimester women. Four (100%) first trimester women, 46 (74.1%) second trimester and 90 (85.7%) third trimester women were immune against measles. One hundred fifty-five (96.8%) of the women up to 34 years of age and 13 (86.6%) of the women who were 35 years old or more were seropositive for rubella virus. One hundred thirty-two (82.5%) of the women up to 34 years of age and 11 (73.3%) of the women who were 35 years old or more were seropositive for measles virus. The levels of IgG against rubella or measles did not correlate significantly with demographic or obstetric characteristics or with a history of medical diseases, except that anti-rubella IgG correlated significantly with age of the pregnant women and gender of the fetuses (Table 1, Figures 1, 2, 3, 4, 5, and 6 ). For both rubella and measles, there were no significant differences between seronegative and seropositive women in trimester of pregnancy, area of residence, employment status, number of persons in the household, number of fetuses in their current pregnancy, gravidity or reference hospital (Table 2). Logistic regression that included simultaneously all variables with a P value of 0.2 or less for their correlation with anti-rubella or anti-measles IgG level (Table 1) did not show any significant relation. There was no correlation between anti-rubella IgG and anti-measles IgG levels (r = 0.08, P = 0.25).

**Table 1:** Correlation of demographic and obstetric characteristics and history of medical diseases with anti-rubella IgG and anti-measles IgG antibody concentration in pregnant women who sought prenatal care at obstetrics and maternity hospitals affiliated with Shiraz University of Medical Sciences in southern Iran from November 2011 to January 2012*.

Item	Anti-rubella IgG	P value	Anti-measles IgG	P value
Age	−0.18	0.01	0.08	0.26
Occupation	0.07	0.22	0.06	0.26
Area of residence	0.04	0.34	0.03	0.66
Number of persons on household	0.02	0.74	−0.04	0.58
Number of children	−0.06	0.39	0.01	0.83
Years since last pregnancy	−0.12	0.16	−0.03	0.69
Current gravidity	−0.06	0.39	0.07	0.31
Body Mass Index Current pregnancy Before current pregnancy	−0.14 −0. 12	0.06 0.11	0.01 0.02	0.85 0.79
Gestational age	−0.04	0.60	0.01	0.81
Number of abortions or stillborn fetuses	−0.07	0.49	0.05	0.59
Number of fetuses in the current pregnancy	−0.008	0.91	−0.08	0.28
Gender of fetus	0.08	0.00	0.09	0.43
Positive history of disease	0.06	0.31	0.19	0.50
Reference hospital	0.07	0.41	0.02	0.13

* Interval-Interval correlations were calculated with Pearson’s correlation coefficient; Interval-Nominal correlations were calculated with the Eta coefficient.

**Table 2:** Comparison of demographic and obstetric characteristics between pregnant women with protective and nonprotective levels of rubella or measles IgG who sought prenatal care at obstetrics and maternity hospitals affiliated with Shiraz University of Medical Sciences in southern Iran from November 2011 to January 2012*.

Item		Anti-rubella IgG				Anti-measles IgG			
		Protective (>11 IU/mL) n = 168 (96%)	Nonprotective(<9 IU/mL) n = 2 (1.14%)	Statistic	P value	Protective (>11 IU/mL) n = 143 (81.71%)	Nonprotective (<9 IU/mL) n = 17 (9.71%)	Statistic	P value
**Area of residence**	Urban	121 (72.03)	2 (100)	FE*	1.00	103 (72.03)	13 (76.47)	FE	1.00
	Rural	47 (27.97)	0 (0)			40 (27.97)	4 (23.53)		
**Employment status**	Employed	10 (5.95)	0 (0)	FE	1.00	9 (6.30)	1 (5.88)	FE	1.00
	Unemployed	158 (94.04)	2 (100)			134 (93.7)	16 (94.12)		
**Number of fetuses in current** **pregnancy**	Single fetus	157 (93.45)	2 (100)	FE	1.00	134 (93.7)	15 (88.23)	FE	0.33
	Multiple fetuses	5 (2.97)	0 (0)			9 (6.3)	2 (11.77)		
**Gravidity**	1st	66 (39.28)	2 (100)	FE	0.15	53 (37.06)	6 (35.29)	?2† = 0.02	0.88
	2nd ≤	102 (60.72)	0 (0)			90 (62.9 3)	11 (64.71)		
**Trimester of pregnancy** ‡	1st (≤14 weeks)	4 (2.38)	0 (0)	?2 = 0.18	0.91	4 (2.79)	0 (0)	?2 = 3.66	0.16
	2nd (14^+1^ to 28 weeks)	60 (35.71)	1 (50)			46 (32.16)	9 (52.94)		
	3rd (≥28^+1^ weeks)	100 (59.52)	1 (50)			90 (62.93 )	7(41.17)		
**Reference hospital**	Hafez	143 (85.12)	2 (100)	FE	1.00	122 (85.31)	12 (70.58)	?2 = 2.42	0.12
	Zeinabieh	25 (14.88)	0 (0)			21 (14.69)	5 (29.42)		

* Pregnant women with equivocal level of IgG (9–11 IU/mL) were not included in the comparisons.

FE: Fischer’s exact test,

χ2†: Chi-squared test,

‡ In 4 (2.28%) pregnant women, gestational age was not determined.

## Discussion

### Anti-rubella Seroprevalence

The incidence of CRS has been decreasing worldwide due to increasing coverage of rubella vaccination [10], but it remains a threatening and costly disease in regions where pregnant women are not immunized and do not have protective levels of IgG against rubella virus. According to WHO policies, the primary goal of rubella vaccination is to prevent congenital rubella infection and CRS. One of the two approaches to the use of rubella-containing vaccines focuses exclusively on reducing CRS by immunizing adolescent girls or women of childbearing age, or both groups. As rubella control progresses towards elimination, the sensitivity and specificity of surveillance systems should increase. If resources permit, periodic seroprevalence surveys could be used to monitor the impact of an immunization program; these surveys could include the collection of samples from women attending antenatal clinics [2]. In all stages of rubella control, rubella surveillance should be integrated with the measles surveillance system [2].

For Iran as a member of WHO, the prevention and control of measles and rubella is a high priority [3]. Several studies conducted from 1968 to 2002 in different provinces of Iran showed that the immunity level against rubella was from 69.9% to 97% among women aged 15 to 45 years, and from 70 to 94% in pregnant women [9]. In 2002 the Ministry of Health and Medical Education of Iran developed a comprehensive strategy to eliminate measles and rubella [3]. A nationwide MR vaccination campaign was conducted in December 2003, and 33 579 082 people (99%) aged 5 to 25 years were vaccinated. Earlier findings had indicated that 61.9% of the target population was immune and 38.1% were susceptible to rubella before vaccination. After the 2003 MR mass vaccination campaign, 98% of the susceptible group acquired immunity and 2% of them did not acquire immunity to rubella [7]. Another survey showed that after the vaccination campaign, rubella immunity reached 91.0%, 99.6%, 99.6% and 97.0% respectively for the 6–10, 11–15, 16–20, and 20–26 year old age groups. Seropositivity for the rubella virus in the target population was high, especially in women of childbearing age (98.9%), thereby preventing congenital rubella infections [11]. Another serosurvey in 2004 after the 2003 MR mass vaccination campaign showed that >97.4% of the population aged between 5 and 40 years had immunity to measles and rubella [3]. According to one study conducted two months after the 2003 of MR vaccination campaign, the immunity rate in the vaccinated population was 98.5% to 99.5%, and for the population more than 25 years of age this rate was higher than 99.5% [12]. It should be noted that all the studies mentioned above were conducted before or at the most one to two years after the 2003 MR mass vaccination campaign, and they used different laboratory methods and considered different cutoff levels of immunity.

To date, no rubella or measles surveillance system for women of childbearing age has been implemented in Iran, so the proportion of pregnant women at risk of developing CRS or measles complications has not been reliably estimated. Therefore, a decade after the 2003 vaccination campaign, current information about the immunity status of pregnant women against these viruses is lacking. Most women (90%) were among the target group for vaccination in 2003, and the remaining 10%, who are currently 35 years old or older, were 26 years old or older in 2003 and were not vaccinated then [13].

The present study found that the overall immunity rate against rubella among pregnant women is 96%, close to the 97% to 99.6% immunity level detected by other surveys in 2004 [9]. We found that compared to the group younger than 35 years (96.9%), a smaller proportion of pregnant women 35 years old or older were immune to rubella (86.7%). However, this difference was not statistically significant and was not related to rubella vaccination in 2003 in the first group.

The level of rubella seropositivity in pregnant women in this study is similar to studies in some countries [14]–[19], but different from other surveys [20]–[23]. In several studies conducted in Turkey, rubella seropositivity among pregnant women was between 93.8% and 100% [15]–[16], [18]. In Sydney, Australia, 93% of pregnant women were rubella-seropositive [17]. The percentage of pregnant women who were immune to rubella was 89.1% in Taiwan [21] and 87% in Germany [23]. Protective levels of anti-rubella IgG were documented in 72% of pregnant women in Sudan [20] and 53% in Nigeria [22]. Rubella seroprevalence among women of childbearing age was reported as 98% in Iowa (USA) [24], 93% in Cartagena (Colombia) [25], 92.5% in Brazil [26], 91.2% in Argentina [27] and 89.5% in Poland [28].

Univariate analysis showed that the age of pregnant women correlated inversely with anti-rubella IgG level, a finding consistent with the results of other studies [17], [21], [25]–[26], [28]–[29] but in contrast to research that reported a significant positive correlation [27] or lack of correlation between these two variables [16], [24]. However, by multivariate analysis, no association was found between rubella seropositivity as a dependent variable and sociodemographic and obstetric characteristics or history of medical diseases as independent variables. This pattern of associations is similar to that found in other studies [16], [19]–[20], but contrasts with surveys that detected an association between rubella immunity and employment status [25], number of persons per household [14], fewer than four pregnancies [27] and nulliparity [17]. Although the pregnant women we surveyed were in different trimesters of their pregnancies, we found no significant differences in rubella immunity; no other studies appear to have compared seropositivity rates among women in different trimesters. Taking into consideration that 10% of the approximately 1 million women who become pregnant per year in Iran are 35 years old or older [13], and our finding that 13.3% of the pregnant women in this age group were not immunized against rubella, it can be estimated that at least 13 300 pregnant women in Iran are at risk of developing CRS. If we add to this figure the 3.1% of pregnant women younger than 34 years who were rubella-seronegative, the number of pregnant women at risk per year becomes 41 200.

**In conclusion**, because of the high rate of anti-rubella IgG seropositivity in our region, we do not recommend routine anti-rubella IgG screening or rubella catch-up vaccination for all women of childbearing age, but do recommend these measures for women 35 years of age or older before marriage or conception. It should be noted that this is a preliminary regional-level study, and further nationwide surveys with larger population sizes will be needed to determine the need for national supplementary immunization activities or anti-rubella screening among women of childbearing age.

### Anti-measles Seroprevalence

Measles during pregnancy may be severe and is linked to a higher incidence of pneumonitis, respiratory distress, stillbirth, spontaneous abortion, hospitalization and death among pregnant women [4], [30]–[33]. The infants of pregnant women infected with measles have a higher incidence of prematurity, hospital admission and subacute sclerosing panencephalitis [4], [31]–[33]. Measles mortality has decreased in recent years because of higher vaccination coverage, and global measles deaths decreased by 78% from 2000 to 2008 [34]. In Iran, measles vaccination coverage between 1980 and 2005 ranged from 38% to 99%, with sustained high coverage (94% to 99% or higher) during the past decade [3]. However, in response to increased numbers of cases in older age groups during 1996–2002, a nationwide MR vaccination campaign was conducted in 2003 [8]. During 2004–2009, 221 laboratory-confirmed measles cases (<1 case per million population) were detected [8]. After the 2003 MR mass vaccination campaign, measles immunity in Iran reached 80.6% in the 6 to 10-year-old population, 72.7% in children aged 11 to 15 years, 84.9% in the 16 to 20-year-old age group and 87.5% in adults aged 20 to 26 years [11]. However, these levels of immunity for measles were significantly lower than the rates required to achieve 95% coverage or higher, the target required for elimination [11]. A survey in Urmia (northern Iran) showed that measles immunity in the population aged 5 to 25 years increased from 53% before to 72.3% after the 2003 mass vaccination campaign [35]. The present study showed that 81.7% of pregnant women were immune against measles, a figure similar to those in other countries. The rate of immunity among pregnant women was 88% in Iowa (USA) [24] and Argentina reported an 87.5% rate of measles seropositivity among women aged 15 to 49 years [27]. Other studies found immunity to measles in 79% of pregnant women in Germany [23], 80% to 90% of pregnant women in Japan [36] and 88.6% of women of childbearing age in Shanghai (China) [37].

In the present study the geometric mean concentration of anti-measles IgG was 13.8 IU/mL, i.e. much lower than the 814.7 IU/mL concentration in pregnant women in Shanghai [37]. We found that the rate of immunization among pregnant women 35 years old or older was lower (73.3%) than among younger women (82.5%), although the difference was not statistically significant. This finding contrasts with the results of other surveys that found a significant positive correlation [27], [36] or negative correlation between measles seropositivity and age [37]. Other studies have reported that the immunity level against measles decreased in pregnant women compared to nonpregnant women [38]–[39]. Our findings showed that the rate of nonimmunity against measles was 26.7% among pregnant women 35 years of age and older, and 17.5% among pregnant women 34 years of age or younger. This difference implies that 184 200 pregnant women annually in Iran are at risk of developing measles complications. We also found that anti-rubella IgG level did not correlate with anti-measles IgG level; therefore immunity against one of these viruses does not predict immunity against the other virus, as reported in an earlier study [24].

**In conclusion**, we recommend anti-measles antibody screening for all women of childbearing age in our region, and especially in women 35 years of age or older who plan to become pregnant, to identify women who would benefit from vaccination. Another recommendation is catch-up measles vaccination for all women of childbearing age. However, the cost-effectiveness of such strategies should be determined first. For nationwide policymaking, larger scale studies that include a representative sample of the entire population are needed. Each of the interventions should include education for obstetricians, gynecologists and other providers of care to pregnant women as an essential step toward the elimination of rubella and measles among women of childbearing age.

This study had some limitations. We included only pregnant women who were followed at university-affiliated hospitals; women who sought care at private hospitals were not included. However, most pregnant women in Shiraz are seen at university-affiliated hospitals. Another limitation was that the only source of information regarding history of medical diseases was self-reporting by the women; their medical records were not systematically checked to verify this information. The number of first-trimester pregnant women in this study was much lower than the number of women in their second or third trimester. This reflected the fact that women in their first trimester are referred to obstetrics and maternity hospitals much less frequently than women in later stages of pregnancy.
